# Real World Incidence and Etiology of Infectious Complications in Adults With Ph‐Negative Acute Lymphoblastic Leukemia Treated With the Pediatric‐Inspired GIMEMA LAL1913 Program. A Campus All Study

**DOI:** 10.1002/hon.70121

**Published:** 2025-08-11

**Authors:** Patrizia Zappasodi, Ludovica Calabretta, Virginia Valeria Ferretti, Davide Lazzarotto, Nicola Fracchiolla, Marco Cerrano, Cristina Papayannidis, Sabina Chiaretti, Maria Ilaria Del Principe, Valentina Mancini, Monia Lunghi, Fabio Forghieri, Sara Mastaglio, Michelina Dargenio, Crescenza Pasciolla, Carla Mazzone, Beatrice Sani, Fabio Guolo, Marzia Defina, Maria Ciccone, Elisa Roncoroni, Marianna Rossi, Claudia Patricia Tobar Cabrera, Gianluca Martini, Giacomo Riccaboni, Luca Arcaini, Robin Foà, Anna Candoni

**Affiliations:** ^1^ UO Ematologia Fondazione IRCCS Policlinico San Matteo Pavia Italy; ^2^ Dipartimento di Medicina Molecolare Università di Pavia Pavia Italy; ^3^ SSD Biostatistica e Clinical Trial Center IRCCS Fondazione Policlinico San Matteo Pavia Italy; ^4^ Clinica Ematologica ‐ Centro Trapianti e Terapie Cellulari Azienda Sanitaria Universitaria Friuli Centrale Udine Italy; ^5^ U.O. Ematologia IRCCS Ca’ Granda Ospedale Maggiore Policlinico di Milano Milano Italy; ^6^ S.C. Ematologia AOU Città della Salute e della Scienza ‐ Presidio Molinette Torino Italy; ^7^ IRCCS Azienda Ospedaliero‐Universitaria di Bologna Istituto di Ematologia “Seragnoli” Bologna Italy; ^8^ Ematologia Dipartimento di Medicina Traslazionale e di Precisione “Sapienza” Università di Roma Roma Italy; ^9^ Dipartimento di Biomedicina e Prevenzione Università di Rome Tor Vergata Roma Italy; ^10^ Dipartimento di Ematologia ASST Grande Ospedale Metropolitano Niguarda Milano Italy; ^11^ Divisione di Ematologia Dipartimento di Medicina Traslazionale AOU Maggiore della Carità Università del Piemonte Orientale Novara Italy; ^12^ Dipartimento di Scienze Mediche e Chirurgiche Clinica Ematologica Azienda Ospedaliero Universitaria di Modena UNIMORE Modena Italy; ^13^ Unità di Ematologia e Trapianto di Midollo Osseo Istituto San Rafaele Milano Italy; ^14^ S.C. Ematologia Unità Trapianti Cellule Staminali Ospedale Vito Fazzi Lecce Italy; ^15^ UO Ematologia con Trapianto ‐ Azienda Ospedaliero‐Universitaria‐Consorziale Policlinico di Bari Bari Italy; ^16^ Ematologia Dipartimento di Medicina Ospedale S. Eugenio Roma Italy; ^17^ SCDU Ematologia e Terapie Cellulari AO Ordine Mauriziano Torino Italy; ^18^ Dipartimento di Medicina Interna (DiMI) Clinica Ematologica Università degli Studi di Genova IRCCS Policlinico San Martino Genova Italy; ^19^ UOC Ematologia Azienda Ospedaliero Universitaria Senese Siena Italy; ^20^ UO Ematologia Dipartimento di Onco‐Ematologia Azienda Ospedaliero‐Universitaria Arcispedale S. Anna Ferrara Italy

**Keywords:** acute lymphoblastic leukemia, GIMEMA LAL1913, infections, real life

## Abstract

Infections often complicate pediatric‐inspired treatments for adult Philadelphia‐negative acute lymphoblastic leukemia (Ph‐ ALL). Literature data on these complications are difficult to interpret due to the heterogeneity of types of infections analyzed or patients and treatment characteristics. A deeper insight on the infections occurring in the real life in uniformly treated ALL patients is lacking. This study investigated infectious complications in 240 newly diagnosed adult Ph‐ ALL patients treated in the real life according to the GIMEMA LAL1913 protocol by 18 Italian centers participating in the Campus ALL network. Incidence, etiology of microbiologically documented infections and invasive fungal infections (IFI) and mortality for infection were determined. Potential risk factors and the prophylactic strategies used during the first chemotherapy course (C1) were analyzed. Of 240 patients, 145 (60%) experienced at least one infectious episode, with bacterial infections being the most common (74.3%), followed by viral (13.9%), fungal (10.1%), and Pneumocystis jirovecii (1.7%) infections. The blood stream was the most involved site, pneumonia occurred in 14.6% of cases, half of which being fungal. Infections were prevalent during C1, affecting 40.5% of patients; IFI occurred in 12.5% of patients, most of them in C1. Risk factors for infections included older age (≥ 55 years and particularly > 65 years) and comorbidities only for IFI. The mortality rate for infection was 3.3%. Antibacterial, antiviral, antifungal, and anti‐PJ prophylaxis were variably administered and did not associate with a significant reduced infection rate. In conclusion, the rate of infectious complications in the real life of adult Ph‐ ALL patients treated with a pediatric‐inspired intensive regimen is high, mainly during induction and mostly bacterial, particularly in the bloodstream, with a high IFI rate. Older age, mainly over 65 years, is a risk factor for all types of infection. The antimicrobial prophylaxis was not associated to a reduced risk of infection.

## 1 Introduction

Besides better outcomes of pediatric‐derived treatments yielded for newly diagnosed adult Philadelphia‐negative acute lymphoblastic leukemia (Ph‐ ALL) [1, 2, 3], infectious complications are frequent, favored by mucosal barriers damage, the more severe and prolonged neutropenia alongside a more profound immunosuppression [4, 5]. Bacterial infections, particularly bloodstream infections (BSI), are frequently reported in adult populations with a variable incidence, ranging from 20% to 50% [6]. Invasive fungal infections (IFI) also represent a worrying treatment complication, reported with a variable incidence both in pediatric ALL ranging from 0.01% to 22% [7, 8] and in adult ALL with incidence from 6.5% to 11.7% [5, 9, 10]. However, the epidemiologic data is heterogeneous, due to large differences in chemotherapy regimens, steroids dosage and duration or prophylaxis administration. No clear prophylactic recommendations are available in ALL: antibiotic prophylaxis is often heterogeneous and not investigated in prospective randomized trials. The only randomized, placebo controlled trial on the effectiveness of prophylactic Lyposomal Amphotericin B (L‐AMB) given during the induction phase in adults Ph‐ ALL, failed to demonstrate a significant advantage in preventing fungal infections [11].

After the completion of the large multicenter GIMEMA LAL1913 clinical trial [12] employing a pediatric‐inspired chemotherapy program for adult Ph‐ ALL patients, the centers participating in the Campus ALL network in Italy adopted the same treatment scheme in the real‐life setting. Lazzarotto et al. collected clinical data of 421 patients and reported the results of their outcome. Although without details, the authors reported bacterial infections as the most common complication and the first induction course (C1) as the most burdened treatment phase [13].

In order to deepen our knowledge on the incidence and type of infections and to describe the prophylactic approaches adopted, we analyzed more in detail data on the infections that occurred during treatment of a newly diagnosed multicenter population of Ph‐ ALL uniformly treated in the real life by the Campus ALL network.

## 2 Material and Methods

We collected data regarding infections of 240 consecutive adult patients with newly diagnosed Ph‐ ALL, treated in the real‐life according to the GIMEMA LAL1913 protocol [12] between September 2016 and December 2022 [13] by 18 Italian centers participating in the Campus ALL network. The objectives of this study were (1) to define incidence and etiology of the infectious complications, (2) to determine the phase of treatment more affected by infections, (3) to assess the mortality due to infections, (4) to describe the prophylactic approaches used during C1 and to assess their impact on the occurrence of infections.

GIMEMA LAL1913 program [13], as shown in the Supporting Information S1: Table 1, consisted in 8 courses of chemotherapy (C1‐C8), did not include immunotherapy and steroids were widely used at high dose, specifically in C1. From complete remission onwards, serial determinations of immunoglobulin plasma levels, with Ig administration (0.4 g/kg IV) for concentration below 0.5 g/dL were recommended.

All documented bacterial infections requiring intravenous treatment (classified as CTCAE.5 grade three or higher) and IFI classified as proven, probable and possible, according to the European Organization for Research and Treatment of Cancer (EORTC) and the Mycoses Study Group Education and Research Consortium (MSG) definitions were included [14]. Febrile events without microbial identification, defined as fever of unknown origin (FUO), were separately analyzed. Bacteremia by coagulase‐negative staphylococci (CoNS) and by Corynebacteria was considered only when supported by at least two positive blood cultures. We excluded non‐IFI and the cases of colonization by multidrug‐resistant bacteria. Recurrent infections caused by the identical etiologic agent and arising in the same patient were considered as one event.

We collected data about the prophylactic treatments administered according to the policy of each center during C1, reported as the most affected course by infectious complications [13]. The incidence of Pneumocystis jiroveci (PJ) and the impact of specific prophylaxis were analyzed separately from fungal events due to the different prophylaxis utilized.

The study was approved by the Ethics Committee of the coordinating center‐Dpt of Hematology University of Udine‐IT and for all participating centers.

### 2.1 Statistical Analysis

Qualitative variables were described as counts and percentage of each category. Quantitative variables were summarized as median and interquartile range (IQR). The association between two qualitative variables was assessed via the Fisher's exact test. The comparison of a quantitative variable between two groups of patients was evaluated by the Mann‐Whitney test for independent data.

To evaluate the association between the use of prophylaxis and the onset of infections during C1, univariable and bi‐variable generalized linear models for binomial family were carried out. Results from these models are reported in terms of risk‐ratio (RR) or risk‐difference (RD) with 95% confidence interval (95% CI). Standard errors were estimated using clustered the Sandwich estimator to allow for intra‐center correlation. In the bi‐variable models the effect of prophylaxis was adjusted for gender, age and comorbidities. Two‐sided type I error was set at 5%. All statistical analyses were performed using Stata 18 (StataCorp.2023. Stata Statistical Software: Release 18. College Station,TX:StataCorpLLC).

## 3 Results

The characteristics of the 240 patients are summarized in Table 1. Median age at diagnosis was 41 years (IQR: 27–51 years) with 9 subjects (3.7%) ≥ 65 years. Among patients with at least one comorbidity (38.6%), cardiologic and endocrinologic diseases were the most frequently reported. We documented 296 infectious events, with 145/240 patients (60.4%) experiencing at least one infectious episode. Infections are described globally (C1‐C8) and during C1 in Table 2. Overall, there were 220/296 (74.3%) bacterial events, 30/296 (10.1%) fungal, 41/296 (13.9%) viral and 5/296 (1.7%) PJ events. Further details on the incidence and etiology of infections are provided in Table 3, Table 4, and Table 5. Most patients who experienced an infection had only one episode, 56 patients (23.3%) had multiple different bacterial infections (Supporting Information S1: Table 2). Infectious complications were prevalent in C1, with 98 patients (40.8%) having a total of 147 events. Throughout the other courses, patients had fewer events: after C1, C6 was mostly burdened by infections (Supporting Information S1: Figure 1). Overall, we observed 44 pulmonary infections (14.9% of all infectious events): 16 bacterial, 21 fungal, 2 viral and 5 due to PJ. In C1, we recorded 30/44 cases of pneumonia, 4 of which being bacterial, 19 fungal, 2 viral and 5 due to PJ.

**TABLE 1:** Characteristics of the 240 studied patients.

Sex, *n* (%)	
Females	94 (38.6%)
Males	146 (61.4%)
Age at diagnosis (years), median (IQR)	41 (27–51)
Age at diagnosis, *n* (%)	
< 55 years	190 (79.2%)
≥ 55 years	50 (20.8%)
Comorbities, *n* (%)	
No	140 (61.4%)
Yes	88 (38.6%)
Number of comorbidities, *n* (%)	
0	140 (61.4%)
1	63 (27.6%)
≥ 2	25 (11%)
Type of comorbidities, *n* (%)	
Cardiologic	20/228 (8.8%)
Endocrinologic/rheumatologic	21/228 (9.2%)
Metabolic syndrome	16/228 (7.0%)
Gastroenterologic	17/228 (7.5%)
Infections	13/228 (5.7%)
Psychiatric	10/228 (4.4%)
Previous neoplasia	9/228 (3.9%)
Pulmonary	5/228 (2.2%)
Hematologic	5/228 (2.2%)
Nephrologic	4/228 (1.8%)
Neurologic	2/228 (0.9%)

Abbreviation: IQR: Interquartile Range.

**TABLE 2:** Incidence and etiology of infection events, globally and in cycle 1 (C1), in 240 patients.

	Overall (*N* = 296)	C1 (*N* = 147)
Bacterial events, *n* (%)	220/296 (74.3%)	92/147 (62.6%)
Viral events, *n* (%)	41/296 (13.9%)	22/147 (15%)
Fungal events, *n* (%)	30/296 (10.1%)	28/147 (19%)
Pneumocistis jiroveci events, *n* (%)	5/296 (1.7%)	5/147 (3.4%)

**TABLE 3:** Bacterial infections classified according to the etiological agent and the site of infection, globally and in C1 only.

	Overall (C1‐C8)	C1 only
Bacterial infection events, *n* (%)	220/296 (74,3%)	92/147 (62.6%)
Gram+ species, *n* (%)	98/220 (44.5%)	47/92 (51.1%)
*Streptococcus* spp	6/220 (2.7%)	6/92 (6.5%)
*Staphylococcus* spp	46/220 (21%)	16/92 (17.4%)
*Enterococcus* spp	12/220 (5.5%)	10/92 (10.9%)
*Clostridium* spp	10/220 (4.5%)	5/92 (5.4%)
*Micrococcus* spp	4/220 (1.8%)	2/92 (2.2%)
Other	10/220 (4.5%)	3/92 (3.3%)
Unidentified	10/220 (4.5%)	5/92 (5.4%)
Gram‐ species, *n* (%)	122/220 (55.5%)	45/92 (48.9%)
*Klebsiella* spp	27/220 (12.3%)	12/92 (13%)
*Pseudomonas* spp	20/220 (9.1%)	9/92 (9.8%)
*Escherichia* spp	55/220 (25%)	16/92 (17.4%)
*Enterobacter* spp	7/220 (3.2%)	4/92 (4.3%)
*Acinetobacter* spp	4/220 (1.8%)	3/92 (3.3%)
Other spp	9/220 (4.1%)	1/92 (1.1%)
Sites of bacterial infections^a^		
Bloodstream	172 (78.2%)^b^	68 (73.9%)
Urinary tract	18 (8.2%)	9 (9.8%)
Skin and soft tissues	20 (9.1%)	9 (9.8%)
Lungs	16 (7.3%)	4 (4.3%)
Gastrointestinal tract	(5%)	6 (6.5%)

*Note:* During all treatment courses, global infectious events were 296, in C1 they were 147.

^a^ Due to the presence of infectious agents isolated in multiple sites, the total percentage is not 100.

^b^ Catheter related infections were 28 out of 172 bloodstream infections (16%).

**TABLE 4:** Fungal infections classified according to the etiological agent and the site of globally and in C1 only.

	Overall C1‐C8	C1 only
Fungal infections events, *n* (%)	30/296 (10,1%)	28/147 (19%)
*Aspergillus* spp	11/296 (3.7%)	9/147 (6.1%)
*Candida* spp	4/296 (1.3%)	4/147 (2.7%)
*Mucor* spp	1/296 (0.3%)	1/147 (0.7%)
*Fusarium* spp	1/296 (0.3%)	1/147 (0.7%)
*Criptococcus* spp	1/296 (0.3%)	1/147 (0.7%)
*Geotrichum* spp	1/296 (0.3%)	1/147 (0.7%)
Sites of fungal infection		
Lungs	21/30 (70%)	19/28 (67.9%)
Bloodsteam	6/30 (20%)	5/28 (17.8%)
Liver and spleen	3/30 (10%)	3/28 (10.7%)
Sinuses	1/30 (3.3%)	1/28 (3.6%)

*Note:* During all treatment courses, global infectious events were 296, in C1 were 147.

**TABLE 5:** Viral infections classified according to the etiological agent globally and in C1 only.

	Overall (C1‐C8)	C1 only
Viral infections events, *n* (%)	41/296 (13.9%)	22/147 (14.9%)
Herpes simplex virus 1	13/296 (4.4%)	12/147 (8.2%)
SARS‐CoV2	14/296 (4.7%)	6/147 (4.1%)
Influenza A	6/296 (2%)	1/147 (0.7%)
Influenza B	1/296 (0.3%)	1/147 (0.7%)
Parvovirus B19	1/296 (0.3%)	1/147 (0.7%)
Ebstein‐Barr virus	1/206 (0.3%)	1/147 (0.7%)
Cytomegalovirus	1/296 (0.3%)	0
Rhinovirus	1/296 (0.3%)	0
Rotavirus	1/296 (0.3%)	0
RSV	1/296 (0.3%)	0
Parainfluenzae	1/296 (0.3%)	0

*Note:* During all treatment courses, global infectious events were 296, in C1 were 147.

Univariable analysis showed age as a risk factor for the development of infections during the entire treatment: patients ≥ 65 years had a significant higher risk of global infections [RR = 1.51 (95% CI: 1.18–1.92) *p* = 0.001], bacterial [RR = 1.51 (95% CI: 1.08–2.12) *p* = 0.016] or fungal infections [RR = 5.14 (95% CI: 2.76–9.55) *p* < 0.001]. Patients ≥ 55 years had only an increased risk of fungal infections [RR 2.69 (95% CI 1.30–5.56), *p* < 0.007]. While gender did not have any significant impact, comorbidities impacted on the development of fungal [RR 2.86 (95% CI 1.74–4.72), *p* < 0.001] and viral events [RR 1.89 (95% CI 1.15–3.10), *p* = 0.012].

Considering C1, age confirmed to be a significant risk factor for infections both in patients ≥ 55 years who had an increased risk of global infections [RR = 1.73 (95% CI: 1.3–2.3) *p* < 0.001], and in those ≥ 65 years for all types of infection [RR 2.32 (95% CI 1.8–3), *p* < 0.001], bacterial [RR 2.31 (95% CI 1.61–3.31), *p* < 0.001], fungal [RR 5.61 (95% CI 2.94–10.67), *p* < 0.001], viral [RR 4.35 (95% CI 1.46–13), *p* = 0.008] and of pneumocystosis [RR 6.17 (95% CI 0.82–46.47), *p* = 0.077].

More than one third of patients (87, 36.2%) experienced a FUO, with 142 recorded events. Fifty‐seven of them occurred in C1 and involved 57 subjects. Globally, 49 patients (20.4%) never developed infections or FUO during the entire treatment, while in C1 92 patients did (38.3%).

Thirteen patients died during the treatment. Infectious complications were the most common cause of death (8/13, 61.5%) with a mortality rate of 3.3%, mainly in C1 (6/8 patients). In detail, 4/8 died of fungal pneumonia, 3/8 had a BSI and 1/8 had a septic shock.

### 3.1 Bacterial Infections

Throughout treatment, we observed grade 3 or higher 220 bacterial infections involving 127/240 patients (52.9%). Most infections were caused by gram‐negative (G‐) bacteria (55.5%), whereas gram‐positive (G+) were responsible for 44.5% of the reported events.

During C1, we recorded 92/147 bacterial complications (62.6%) in 74 of the 98 patients who had infections in C1 (75.5%). Gram‐ and G+ bacteria were equally distributed (Table 3). The most frequent site was the bloodstream, followed by skin and soft tissues (SSTIs), urinary tract (UTIs) and less commonly gastrointestinal tract (GIT).

### 3.2 Fungal Infections

IFI were recorded in 30/240 patients (12.5%), 28 of them in C1. We diagnosed 8 proven, 19 probable and 3 possible IFI. The most frequently involved sites were the lung followed by the bloodstream, liver, spleen, and sinuses. We observed 21 fungal pneumonias, 19 of which in C1. Of the identified species, 14 were molds and 5 yeasts. The involved sites and the etiologic agents are detailed in Table 4.

### 3.3 Viral Infections

A total of 41 viral infections were recorded in 37/240 patients (15.5%). Of note, 12 patients had a SARS‐CoV2 infection, half of which occurred in C1. Two viral pneumonias were caused by the Epstein‐Barr virus and by the Herpes Simplex virus. Cutaneous or mucosal Herpes virus 1 reactivations were the most frequent viral events (13/41, 31.7%) specifically in C1. The description of the viral infections is detailed in Table 5.

### 3.4 Antimicrobial Prophylaxis

Antibiotic, antimycotic, antiviral and anti‐PJ prophylaxis was administered based on the antimicrobial policy of each participating center (Table 6). During C1, 120 subjects (50%) received antibacterial prophylaxis, mainly with fluoroquinolones, 197 patients (82%) received antiviral prophylaxis with acyclovir or valacyclovir, 49 patients (20.4%) received anti‐PJ prophylaxis with trimethoprim/sulfamethoxazole, and antimycotic prophylaxis was administered to 150 patients (62.5%). Among antifungals used, micafungin was administered in most patients (52; 34.7%) followed by triazoles (46; 30.7%), and L‐AMB (37; 24.7%). Non‐antimold prophylaxis was administered to 15 patients (10%).

**TABLE 6:** Distribution of the antimicrobic prophylaxis during C1.

Prophylaxis	Yes	No
Antibacterial, *n* (%)	120/240 (50%)	120/240 (50%)
Fluoroquinolones, *n* (%)	117/120 (97.5%)	
Amoxicillin, *n* (%)	3/120 (2.5%)	
Antiviral, *n* (%)	197/240 (82%)	43/240 (18%)
Acyclovir, *n* (%)	179/197 (909%)	
Valacyclovir, *n* (%)	18/197 (9.1%)	
Antifungal, *n* (%)	150/240 (62.5%)	90/240 (37,5%)
L‐AMB^a^, *n* (%)	37/150 (24.7%)	
Triazoles, *n* (%)	46/150 (30.7%)	
Echinocandin, *n* (%)	52/150 (34.6%)	
Fluconazolo, *n* (%)	15/150 (10%)	
Anti Pneumocistis Jiroveci^b^	49/240 (20.4%)	89/240 (37.1%)
Sulf/Trim^c^, *n* (%)	49/49 (100%)	

^a^ L‐AMB: Liposomal Amphotericin B (L‐AMB) 50 mg alternate days.

^b^ For 102 patients, information regarding anti PJ prophylaxis was not provided.

^c^ Sulf/trim = Sulfamethoxazole/trimethoprim.

Antibacterial prophylaxis was not associated with a reduced risk of developing a bacterial infection [RD –0.01 (95% CI: −0.16 to 0.14), *p* > 0.9], also after adjusting for age [RD –0.02 (95% CI: −0.15 to 0.12), *p* = 0.803] or for comorbidity [RD –0.04 (95% CI: −0.17 to 0.10), *p* = 0.597]. No significant differences were seen in the rates of Herpes virus 1 reactivations between patients receiving or not specific prophylaxis [5.1% versus. 4.6%, RD 0.01 (95% CI: −0.08–0.09), *p* = 0.9], also after adjustment for age [RD 0.05 (95% CI: −0.02–0.12), *p* = 0.195] and for comorbidities [RD –0.00 (95% CI: −0.08 to 0.08), *p* > 0.9]. Trimethoprim/sulfamethoxazole did not reduce the risk of developing a PJ infection [RD –5.6% (CI: −11%–1.7%), *p* = 0.161]. It was administered to 49/240 patients (20.4%), while 89 (37.2%) did not receive it; for 102 patients this information was lacking. The 5 cases of PJ pneumonia occurred in C1 in patients who had not received anti‐PJ prophylaxis.

Lastly, regarding fungal infections incidence, in a setting of a heterogeneous prophylaxis policy, no significant differences were found between patients who received antifungal prophylaxis and those who did not: 16/150 patients (10.7%) had a mycotic infection despite prophylaxis, whereas 12/90 (13.2%) had a mycosis without prophylaxis [RD –0.02 (95% CI: −0.10 to 0.05), *p* = 0.542]. These results were confirmed also in bivariate analysis adjusting for age and comorbidities.

## 4 Discussion

The aim of this study was to describe the incidence and etiology of infectious complications occurring during the frontline treatment of adult Ph‐ ALL patients homogeneously treated in the real life according to the pediatric‐inspired GIMEMA LAL1913 protocol. The relevance of infectious complications is documented by the Italian study GIMEMA LAL1913 that reported infections as the main cause of death, also in the remission phase [12]. Also the large Italian retrospective Campus ALL study analyzing real life data from 421 Ph‐ ALL patients treated with the same therapeutic program reported infectious complications mostly BSI and in the first course; however, no further data were reported [13]. Therefore, additional information was thereafter obtained from the participating centers and the available data on the infectious complications that occurred in 240 Ph‐ ALL patients have been analyzed and are hereby described. We found a high incidence of infections occurring in more than half of the patients (59.8%) and confirmed that C1 was the most involved treatment phase, with 98 patients (40.8%) having one or more infections. As reported by other authors [6], bacterial events were prevalent over fungal or viral, affecting 52.9% of patients and consisting in BSI in the majority of events. Literature data from different series is often difficult to interpret due to the broad variability of therapeutic schedules employed and to the different accuracy in considering only documented infective events. In this regard, the Italian SEIFEM prospective study by Di Blasi et al. [6] reported an overall incidence of infections and febrile events of 53%, 19.4% of which being of bacterial nature. This incidence is lower than the one hereby observed, probably due to the different treatments received, to the inclusion also of Ph + ALL patients or to the inclusion of a third of cases in consolidation. Indeed, in our analysis, consolidation was much less complicated by infections than the induction phase. Regarding bacterial infections, we found a high incidence of BSIs, comparable to that observed on the above study. A retrospective French study focusing on BSI and IFI in children, adolescents and young adults (AYA) ALL also showed a high incidence of BSI (51% in AYA patients), with an equal distribution of G+ and G− infections [15], in line with our observation.

Although less frequent than bacterial events, IFI occurred in 12.5% of patients, mainly during C1. While this incidence is higher than previously reported [5, 6, 16], other authors found a high percentage of IFI: Trimbour et al. [15] reported an incidence of 22% of IFI in AYA patients, significantly higher than in children even though also pediatric patients experience more fungal complications when treated intensively [16]. A rate of 15% of invasive pulmonary aspergillosis was reported in the real‐life setting of ALL adults patients receiving the GIMEMA LAL1913 scheme or similar [17]. We confirm that molds are the main responsible of IFI and that lungs are the prevalent site of infection; importantly, IFI are confirmed as the infections primarily responsible of death. Viral infections have a limited impact and one third of them were due to SARS COV2. The time of data collection included the pandemic period; thus, the incidence of viral infections resulted somewhat increased.

Regarding the phase of treatment in which infectious complications were recorded, C1 was the most involved by all different types of infective agents. Indeed, 98 of the 145 infected patients (67.6%) suffered from infections in C1. Furthermore, C1 resulted at high risk for IFI, occurring in 28 of the 30 patients (93%) who experienced fungal complications, and it was the only course with pneumocystosis diagnosed. Importantly, even if mortality rate for infections was low, almost all deaths for infection occurred in C1.

After C1, infections decreased significantly. However, special attention should be paid to C6, where the incidence was 24%. Information on the depth and the duration of the neutropenia caused by course 6 is not available; although C6 differs from C2 only for the inclusion of Vincristine, it is reasonable to hypothesize that hematologic toxicity increases during treatment and tolerability necessarily decreases.

Age demonstrated to be the only factor associated with the risk of infections in patients aged ≥ 55 years and consistently in those over 65. In order to limit toxicity with increasing age, the therapeutic scheme provided an age‐based dose adjustment for some drugs (Idarubicin, PEG‐Asparaginase and Methotrexate) in patients 55 years or older. It has not been reported if the dose adjustment by age was useful to mitigate hematologic or extra‐hematologic toxicity [13]; however, we found a higher impact of infections in patients aged 55 or older. Although only 9 patients (3.9%) were 65 years or more, the higher incidence of infections also for each type ‐ bacterial, fungal or viral ‐ suggests that the GIMEMA ALL1913 program should be administered with caution in patients older than 65 years.

The studied population had a high comorbidity rate (38.6%) but this did not influence the development of infections, except for fungal infections, suggesting that a careful management of patients during all treatment can overcome the negative impact of concomitant diseases. Fungal infections remain the complication that primarily requires a strict monitoring and a prompt intervention.

Regarding the anti‐infective prophylaxis, no clear recommendations are available for ALL patients due to the lack of a proven beneficial effect of a given prophylaxis program. Literature data focused on studies with different endpoints and the only randomized trial on the efficacy of anti‐mold prophylaxis in induction failed to demonstrate a significant benefit of the prophylaxis compared to patients without prophylaxis [10]. In the real life, anti‐infective prophylaxis is very heterogeneous and usually depends on the local infectious epidemiology and the center's choice and policy. While acyclovir is widely used as antiviral agent and fluoroquinolones are the most widely used antibacterial drugs, due to their broad spectrum of activity and their good safety profile, the choice of the optimal antifungal therapy is more difficult. In fact, while fluconazole is largely ineffective due to molds' intrinsic resistance, triazoles interaction with alkaloids limits their use in induction and echinocandins are well tolerated but exert only a fungistatic activity toward molds. In the Italian landscape described in this study, all three classes of antifungals were used without a clear prevalence of one over the other and no patients' characteristics were associated with the choice of carrying out or not a prophylaxis or to administer a specific antifungal drug. Our analysis did not show a reduced incidence of infections in patients receiving prophylaxis. However, this study only describes the practical approach of different Italian centers and, due to its observational nature, we cannot draw definitive conclusions about the effectiveness of the antibacterial, antifungal or antiviral prophylaxis. Regarding anti‐PJ prophylaxis, although pneumocystosis is rare, all the cases observed in this study were diagnosed in induction. Therefore, it seems advisable to start specific prophylaxis already from C1.

## 5 Conclusions

This real‐life experience shows that infectious complications, particularly bacterial infections, are frequent during the induction of Ph‐ ALL adults and that age contributes to increase the risk. This is relevant in the current era, when even older patients are offered active treatments. Moreover, fungal infections remain a life‐threatening complication occurring with a non‐negligible incidence and with an impact on mortality.

## Author Contributions

P.Z. designed the study, wrote and revised the paper. L.C. wrote the paper and collected data, V.V.F. performed the analysis, D.L., F.F., L.A., R.F. and A.C. collected data and contributed to the revision of the paper. All other authors collected data and contributed to the final approval of the manuscript.

## Conflicts of Interest

The authors declare no conflicts of interest.

## Peer Review

The peer review history for this article is available at https://www.webofscience.com/api/gateway/wos/peer-review/10.1002/hon.70121.

## Supporting information


Supporting Information S1

## Data Availability

The data that support the findings of this study are available on request from the corresponding author. The data are not publicly available due to privacy or ethical restrictions.
