# Supplementary material for: Real World Incidence and Etiology of Infectious Complications in Adults With Ph‐Negative Acute Lymphoblastic Leukemia Treated With the Pediatric‐Inspired GIMEMA LAL1913 Program. A Campus All Study
Source: Hematol Oncol. 2025 Aug 11;43(5):e70121. doi: 10.1002/hon.70121 (PMC12337620; doi:10.1002/hon.70121)
Supplement: Supplementary file 1 — Supporting Information S1 [file HON-43-e70121-s001.docx]

**Supplementary Figure 1**. Percentage of patients with complications in each course of treatment.


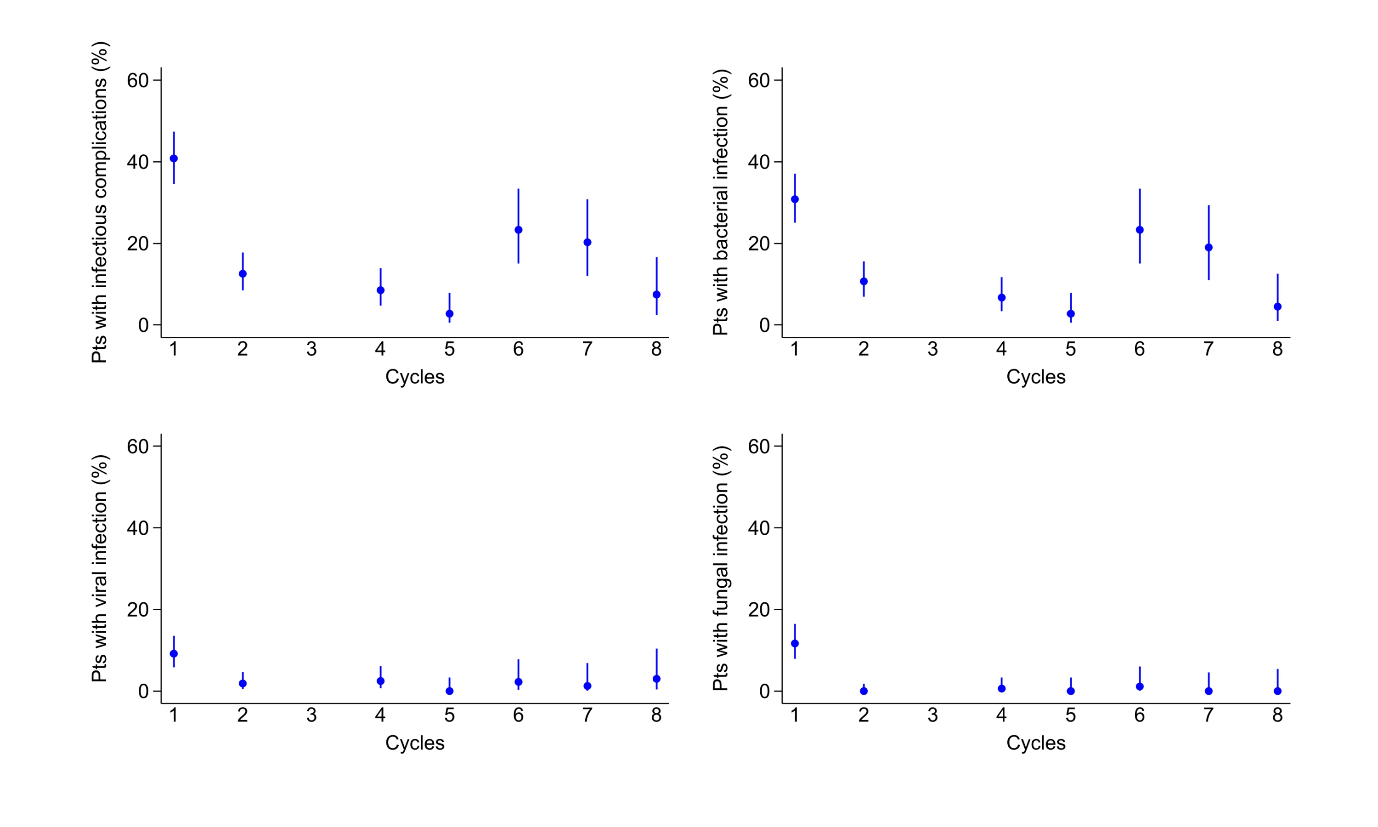


| **Courses** | **N° of patients** | **N° of patients with infection** | **N° of patients with bacterial infection** | **N° of patients with viral infection** | **N° of patients with fungal infection** |
| --- | --- | --- | --- | --- | --- |
| **Course 1** | 240 | 98 (40.8%) | 74 (30.8%) | 22 (9.2%) | 28 (11.7%) |
| **Course 2** | 215 | 27 (12.6%) | 23 (10.7%) | 4 (1.9%) | 0 (0.0%) |
| **Course 3** | 206 | 38 (18.5%) | 35 (17.0%) | 3 (1.5%) | 0 (0.0%) |
| **Course 4** | 164 | 14 (8.5%) | 11 (6.7%) | 4 (2.4%) | 1 (0.6%) |
| **Course 5** | 109 | 3 (2.8%) | 3 (2.8%) | 0 (0.0%) | 0 (0.0%) |
| **Course 6** | 90 | 21 (23.3%) | 21 (23.3%) | 2 (2.3%) | 1 (1.1%) |
| **Course 7** | 79 | 16 (20.3%) | 15 (19.0%) | 1 (1.3%) | 0 (0.0%) |
| **Course 8** | 67 | 5 (7.5%) | 3 (4.5%) | 2 (3.0%) | 0 (0.0%) |

**Supplementary Table 1.** Treatment protocol

| **Treatment phase** | **Drugs** | **Dosing** | **Days** |
| --- | --- | --- | --- |
| **Prephase** | Prednisone  Cyclophosphamide | 20 mg/m^2^ q12h  300 (200 if age >55) mg/m^2^ | -5 to -1  -3 to -1 |
| **Course 1 (C1)** | Idarubicin  Vincristine  Dexamethasone  Pegaspargase  IT prophylaxis | 12 (9 if age >55) mg/m^2^  1.4 mg/m^2^ (max. 2 mg)  5 mg/m^2^ q12h  2000 (1000 if age >55) UI/ m^2^ | 1, 2  1, 8, 15, 22  1-5, 15-19  10  1, 15 |
| **Course 2,4,6 (C2, C4, C6)** | Vincristine  Idarubicin  Cyclophosphamide  Dexamethasone  Cytarabine  Pegaspargase  Mercaptopurine  IT prophylaxis | 1.4 mg/m^2^ (max. 2 mg)  12 (9 if age >55) mg/m^2^  1000 mg/m^2^  5 mg/m^2^ q12h  75 mg/m^2^  2000 (1000 if age >55) UI/ m^2^  60 mg/m^2^ | 1,8 (no course 2)  1  1  1-5  2-5  8 (no course 4)  1-10  1 (and 15, course 2) |
| **HD courses 3,7 (C3, C7)** | Methotrexate  Cytarabine | 2500 (B), 5000 (T), 1500 (if age >55) mg/m^2^ over 24 hours  2000 mg/m^2^ | 1  3 ,4 |
| **HD course 5**  **(C5)** | Methotrexate  Pegaspargase  Mercaptopurine | 2500 (B), 5000 (T), 1500 (if age >55) mg/m^2^ over 24 hours  2000 (1000 if age >55) UI/ m^2^  25 mg/m^2^ | 1  3  8-18 |
| **Course 8**  **(C8)** | Vincristine  Idarubicin  Dexamethasone  Cyclophosphamide  Prednisone  IT prophylaxis | 1.4 mg/m^2^ (max. 2 mg)  10 (7.5 if age >55) mg/m^2^  5 mg/m^2^ q12h  300 (200 if age >55) mg/m^2^  20 mg/m^2^ q12h | 1, 8  1, 8  1-5  1-3  8-12  1, 15 |
| **Maintenance courses**  **M1,3,5,7,9,11** | Cyclophosphamide  Mercaptopurine  Methotrexate  IT prophylaxis | 100 mg/m^2^  75 mg/m^2^  15 mg/m^2^ | 1-4  8-28  8, 15, 22  1 (courses 3,5) |
| **Maintenance courses**  **M2,4,6,8,10,12** | Vincristine  Prednisone  Mercaptopurine  Methotrexate  IT prophylaxis | 1 mg/m^2^ (max. 2 mg)  20 mg/m^2^ q12h  75 mg/m^2^  15 mg/m^2^ | 1  1-5  8-28  8, 15, 22  1 (courses 2,4) |
| **Maintenance courses M13-24** | Mercaptopurine  Methotrexate | 75 mg/m^2^  15 mg/m^2^ | 1-28  1, 8, 15, 22 |

Abbreviations: IT, intrathecal

**Supplementary Table 2.** Number of infectious events per patient, overall and in Course 1 (C1)

|  | Overall | C1 |
| --- | --- | --- |
| Patients with infections, n (%) | 145/240 (60.4%) | 98/240 (40.6%) |
| Number of bacterial infections, n (%) | 127/240 (52,9%) | 74/240 (30,8%) |
| 0 | 113/240 (47.1%) | 166/240 (69.2%) |
| 1 | 71/240 (29.5%) | 60/240 (25.0%) |
| 2 | 34/240 (14.2%) | 11/240 (4.6%) |
| ≥3 | 22/240 (9.2%) | 3/240 (1.2%) |
| Number of viral infections, n (%) | 37/240 (15.5%) | 22/240 (9.2%) |
| 0 | 203/240 (84.6%) | 218/240 (90.8%) |
| 1 | 35/240 (14.6%) | 22/240 (9.2%) |
| 2 | 2/240 (0.8%) | 0/240 (0.0%) |
| Number of fugal infections, n (%) | 30/240 (12.5%) | 28/240 (11.7%) |
| 0 | 210/240 (87.5%) | 212/240 (88.3%) |
| 1 | 30/240 (12.5%) | 28/240 (11.7%) |
| Number of Pneumocistis Jiroveci infections, n (%) | 5/240 (2.1%) | 5/240 (2.1%) |
| 0 | 235/240 (97.9%) | 235/240 (97.9%) |
| 1 | 5/240 (2.1%) | 5/240 (2.1%) |

.
